# Supplementary material for: Role of Intraparotid and Neck Lymph Node Metastasis in Primary Parotid Cancer Surgery: A Population-Based Analysis
Source: Cancers (Basel). 2022 Jun 7;14(12):2822. doi: 10.3390/cancers14122822 (PMC9220808; doi:10.3390/cancers14122822)
Supplement: Supplementary file 1 [file cancers-14-02822-s001.zip › cancers-1662316-supplementary.pdf]

Supplementary Materials

Role of Intraparotid and Neck Lymph Node Metastasis in Primary Parotid Cancer Surgery: A Population-Based Analysis

Mussab Kouka, Benjamin Köhler, Jens Buentzel, Holger Kaftan, Daniel Boeger, Andreas H. Mueller, Andrea Wittig, Stefan Schultze-Mosgau, Thomas Ernst, Peter Schlattmann and Orlando Guntinas-Lichius

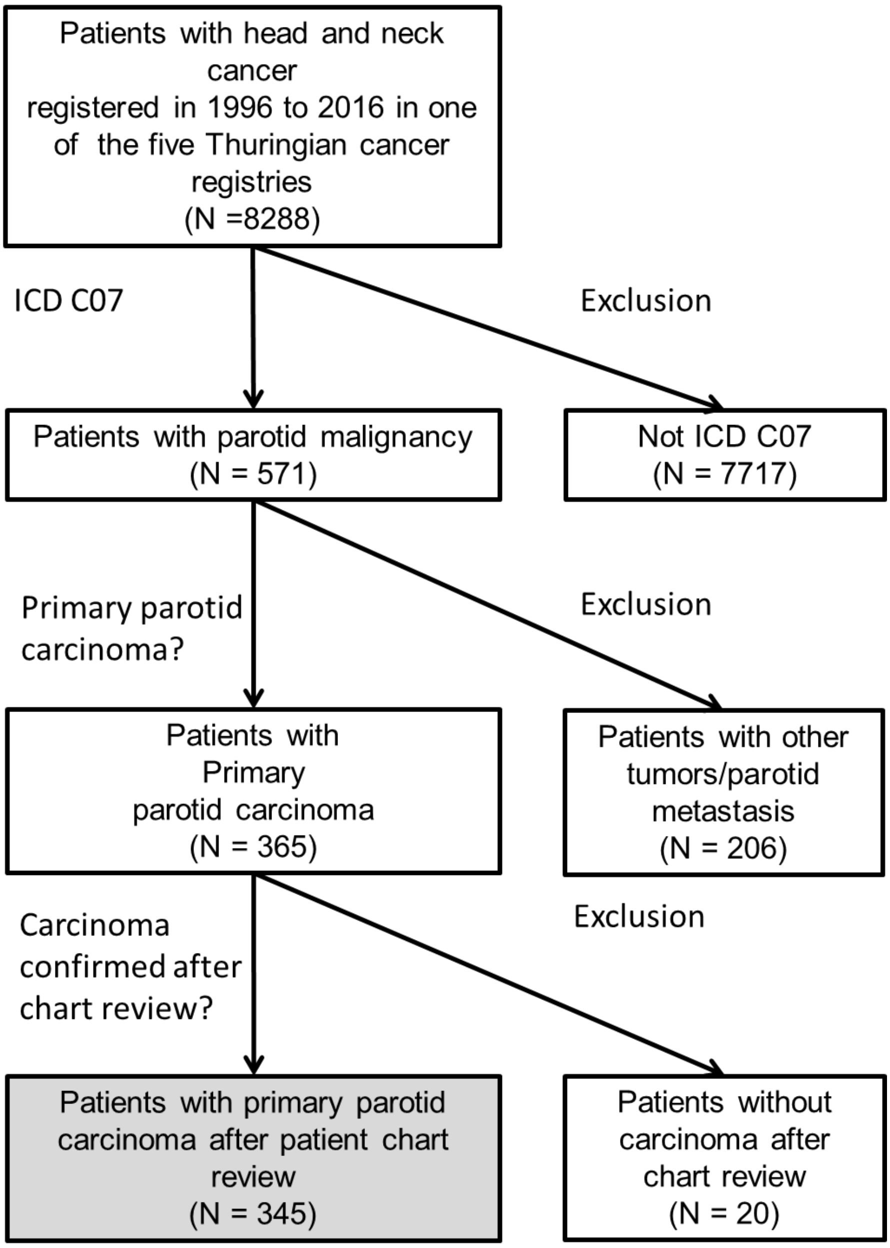

Figure S1. Patients' inclusion and exclusion criteria. Flowchart showing the primary data set and the final data set after data cleansing.

Table S1. Association between intraparotid lymph node examination and clinical parameters.

Parameter	Intraparotid lymph nodes Not examined $n = 173$	Intraparotid lymph nodes Examined $n = 145$	No parotidectomy $n = 27$	p
Gender				0.561
Female	69	65	13	
Male	104	80	14	
Age				**0.001**
<Median 66 years	75	89	9	
≥Median 66 years	98	56	18	
Histology				**0.023**
Carcinoma not specified	19	2	3	
Undifferentiated carcinoma	2	4	0	
Other rare carcinomas	5	4	1	
Squamous cell carcinoma	37	15	8	
Adenocarcinoma	37	35	5	
Adenoid cystic carcinoma	12	21	3	
Mucoepidermoid carcinoma	14	20	2	
Salivary duct carcinoma	5	5	1	
Acinic cell carcinoma	23	17	0	
Epithelial-myoepithelial carcinoma	7	7	0	
Carcinoma ex-pleomorphic adenoma	8	13	3	
Myoepithelial carcinoma	4	2	1	
pT				**0.001**
T1/T2	78	72	8	
T3/T4	56	60	9	
Tx	39	13	10	
N				**<0.001**
N0	71	90	7	
N+	48	39	7	
Nx	54	16	13	
M				**0.001**
M0	152	137	20	
M1	5	2	4	
Mx	16	6	3	
Stage				**<0.001**
I/II	49	62	4	
III/IV	74	68	15	
Unknown	50	15	8	
Parotidectomy*				**<0.001**
Lateral	37	26	NA	
Partial lateral	9	5	NA	
Total	65	99	NA	
Radical	21	15	NA	
Not specified	41	0	NA	
Neck dissection				**<0.001**
No	126	29	26	
Yes	47	116	1	
Radiotherapy				**<0.001**
No	85	82	3	
Yes	88	63	24	
Chemotherapy/Biologicals				**<0.001**
No	159	133	17	
Yes	14	12	10	
Tumor recurrence				0.635

No	145	127	23	
Yes	28	18	4	
Death				**<0.001**
No	100	98	7	
Yes	73	47	20	

* When excluding the cases without parotidectomy. Significant *p*-values (*p* <0.05) in bold.

Table S2. Association of baseline and tumor characteristics on overall survival due to Kaplan–Meier calculations.

**Parameter**	**Categorized**	**2-year OS %**	**5-year OS %**	**Log Rank *p***
All		76.0	64.9	
Gender	Male	72.4	62.8	0.061
	Female	80.9	67.6	
Age, median: 66 years	<Median	85.8	78.8	**<0.001**
	>Median	66.6	50.9	
T classification	T1/T2	81.7	69.5	**0.002**
	T3/T4	70.8	60.3	
N classification	N0	92.5	79.0	**0.005**
	N+	63.7	55.0	
M classification	M0	77.8	66.8	**<0.001**
	M+	28.3	28.3	
UICC stage	I-II	87.3	72.7	**<0.001**
	III-IV	68.5	59.5	
PAR	PAR−	87.6	79.3	**0.009**
	PAR+	54.1	25.7	
PAR, median 3	<Median	80.3	67.2	0.331
	≥Median	87.3	79.7	
PAR+, median 0	≤Median	87.6	78.6	**0.011**
	>Median	73.4	59.5	
PARR, median 0	≤Median	87.6	78.6	**0.011**
	>Median	73.4	59.5	
PARR	<1	85.1	76.5	**0.049**
	≥1	77.4	48.4	
PARLODDS, median −0.70	<Median	90.5	84.7	**0.038**
	>Median	79.7	66.5	
TNOD, median 10 LN	≤Median	89.7	79.5	0.126
	>Median	84.1	72.1	
TNOD, <≥ 12 LN	<Median	90.3	80.7	0.098
	≥Median	81.3	67.8	
TNOD, <≥14 LN	<Median	89.3	79.5	0.128
	≥Median	81.6	68.0	
TNOD, <≥ 16 LN	<Median	88.3	78.8	**0.034**
	≥Median	81.6	64.7	
TNOD, <≥ 18 LN	<Median	88.6	79.3	**0.007**
	≥Median	79.3	60.8	
PNOD, median 0	≤Median	94.9	83.6	**0.001**
	>Median	72.9	54.4	
LNR, median 0	≤Median	88.1	83.6	**<0.001**
	>Median	72.9	54.4	
LODDS, median −1.23	<Median	94.7	84.7	**0.042**
	≥Median	81.4	64.6	
TNOD-PAR, median 11 LN	<Median	83.6	71.4	0.809
	≥Median	86.3	75.6	
PNOD-PAR, median 0	<Median	90.7	80.1	**0.002**

	≥Median	72.6	57.6	
LNR-PAR, median 0	<Median	90.7	80.1	**0.002**
	≥Median	72.6	57.6	
LODDS-PAR, median −1.11	<Median	93.4	85.8	**0.005**
	≥Median	77.0	60.6	
Histology	Adenocarcinoma	78.2	68.4	**<0.001**
	Squamous cell carcinoma	49.4	37.7	
	Acinic cell carcinoma	88.3	82.7	
	Mucoepidermoid carcinoma	88.6	85.3	
	Adenoid cystic carcinoma	94.0	83.6	
	Carcinoma not specified	76.1	69.2	
	Carcinoma ex-pleomorphic adenoma	67.0	47.8	
	Other rare carcinomas	80.0	42.9	
	Epithelial-myoepithelial carcinoma	92.3	82.1	
	Salivary duct carcinoma	53.0	31.8	
	Myoepithelial carcinoma	35.7	35.7	
	Undifferentiated carcinoma	66.7	44.4	
Squamous cell carcinoma	No	89.6	77.6	**0.008**
	Yes	66.3	55.7	
Adenoid cystic carcinoma	No	73.8	63.7	**0.024**
	Yes	94.0	83.6	
Type of parotidectomy	Partial lateral	91.7	73.3	**<0.001**
	Lateral	81.4	74.2	
	Total	76.2	63.6	
	Radical	87.3	76.5	
	Not specified	75.2	68.2	
	No parotidectomy	40.9	24.9	
Neck dissection	No	66.0	55.1	**<0.001**
	Yes	87.2	75.5	
Radiotherapy	No	83.0	73.6	**0.007**
	Yes	69.8	57.2	
Chemotherapy/Biologicals	No	77.0	67.6	**0.012**
	Yes	68.5	44.0	
Years of treatment	1996–2000	74.7	63.8	0.706
	2001–2005	73.6	65.2	
	2006–2011	75.6	60.8	
	2012–2016	83.1	74.7	

OS = overall survival; CI = 95% confidence interval; PAR−= intraparotid lymph nodes without metastasis; PAR+ = intraparotid lymph node metastasis; PARR = intraparotid lymph node ratio; PARLODDS = log odds of positive lymph nodes, parotid; TNOD = number of resected neck lymph nodes, ipsilateral; PNOD = positive neck lymph nodes, ipsilateral; LNR = neck lymph node ratio, ipsilateral; LODDS = log odds of positive lymph nodes, neck, ipsilateral; TNOD-PAR = number of resected of neck and parotid lymph nodes, ipsilateral; PNOD-PAR = positive neck and parotid lymph nodes, ipsilateral; LNR-PAR = neck and parotid lymph node ratio, ipsilateral; LODDS-PAR = log odds of positive lymph nodes, neck and parotid, ipsilateral; LN = lymph node; significant *p*-values ($p < 0.05$) in bold.

Table S3. Cox regression*: independent factors associated with worse overall survival.

Parameter	HR	95% CI lower	95% CI upper	*p*
**Model 2b: Treatment and staging characteristics**				
Neck dissection				
No	1	Reference		
Yes	0.455	0.308	0.672	**<0.001**
Radiotherapy				
No	1	Reference		
Yes	1.150	0.759	1.742	0.510
Chemotherapy/Biologicals				
No	1	Reference		
Yes	1.488	0.899	2.462	0.122
AJCC Stage				
I	1	Reference		
II	2.900	1.467	5.735	**0.002**
III	1.844	0.868	3.916	0.111
IV	4.118	2.197	7.718	**<0.001**
**Model 3b: Traditional staging I**				
T classification				
T1	1	Reference		
T2	3.052	1.636	5.693	**<0.001**
T3	2.640	1.329	5.245	**0.006**
T4	3.603	1.778	7.302	**<0.001**
N classification				
N0	1	Reference		
N1	0.546	0.217	1.373	0.198
N2	1.502	0.958	2.355	0.076
N3	8.857	1.173	66.896	**0.034**
M classification				
M0	1	Reference		
M1	3.388	1.417	8.101	**0.006**
**Model 4b: Traditional staging II**				
T classification				
T1	1	Reference		
T2	3.097	1.663	5.767	**<0.001**
T3	2.656	1.341	5.259	0.005
T4	3.804	1.889	7.657	**<0.001**
N classification				
N0	1	Reference		
N1	0.536	0.213	1.346	0.184
N2	1.641	1.059	2.543	**0.027**
N3	8.207	1.089	61.845	**0.041**
**Model 5b: Alternative staging I**				
T classification				
T1	1	Reference		
T2	2.350	0.808	6.833	0.117
T3	3.916	1.378	11.133	**0.010**
T4	4.734	1.436	15.603	**0.011**
LODDS	1.302	0.817	2.074	0.267
**Model 6b: Alternative staging II**				
T classification				
T1	1	Reference		
T2	2.878	1.117	7.412	**0.029**
T3	3.489	1.354	8.990	**0.010**

T4	5.763	2.104	15.781	**0.001**
LODDS-PAR	1.505	1.065	2.127	**0.020**

LODDS = Log odds of positive lymph nodes, neck, ipsilateral; LODDS-PAR = log odds of positive lymph nodes, neck and parotid, ipsilateral. *The Cox regression models with same but dichotomized parameters is presented in the main text in Table 5. Significant *p*-values ($p < 0.05$) in bold.

Table S4. Cox regression: independent factors associated with worse overall survival; same calculation as in Table 4, but with other references. Adenocarcinoma is now reference for the histologies and total parotidectomy for the parotidectomy techniques. This allows for more robust estimators for Table S5.

Parameter	HR	95% CI lower	95% CI upper	*p*
**Model 1: Patient, tumor and treatment characteristics**				
Age, median: 66 years				
<Median	1	Reference		
>Median	2.91	1.93	4.38	<0.001
Histology				
Adenocarcinoma	1	Reference		
Carcinoma not specified	0.60	0.26,	1.39	0.2
Undifferentiated carcinoma	1.43	0.49,	4.19	0.5
Other subtypes	1.40	0.57,	3.44	0.5
Squamous cell carcinoma	1.78	1.10,	2.90	**0.019**
Adenoid cystic carcinoma	0.50	0.24,	1.03	0.061
Mucoepidermoid carcinoma	0.64	0.29,	1.44	0.3
Salivary duct carcinoma	2.64	1.14	6.12	**0.024**
Acinic cell carcinoma	0.67	0.32	1.38	0.3
Epithelial-myoepithelial carcinoma	0.39	0.14	1.14	0.085
Carcinoma ex-pleomorphic adenoma	1.61	0.7	3.28	0.2
Myoepithelial carcinoma	1.90	0.56	6.45	0.3
Type of parotidectomy				
Total	1	Reference		
Lateral	0.53	0.30,	0.91	**0.022**
Partial lateral	0.59	0.25	1.37	0.2
Extend unclear	0.51	0.27	0.95	**0.035**
Radical	0.85	0.47	1.55	0.6
No parotid surgery	1.66	0.90	3.07	0.10
Neck dissection				
No	1	Reference		
Yes	0.55	0.37	0.82	**0.003**
Radiotherapy				
No	1	Reference		
Yes	1.02	0.69	1.50	0.9
Chemotherapy/Biologicals				
No	1	Reference		
Yes	1.38	0.805	2.36	0.2

HR = hazard ratio; CI = confidence interval; significant *p*-values ($p < 0.05$) in bold.

Table S5. Cox regression: independent factors associated with worse overall survival. All covariates of Table S4 without significance eliminated, but alternative lymph node staging classificators were added. The parotidectomy techniques were also excluded, because its inclusion did not deliver useful results.

Parameter	HR	95% CI lower	95% CI upper	*p*
**Model 2c: Age, histology, treatment, and staging characteristics**				
Age, median: 66 years				
<Median	1	Reference		
>Median	2.69	1.71,	4.25	<0.001
Histology				
Adenocarcinoma	1	Reference		
Carcinoma not specified	0.82	0.33	2.05	0.7
Undifferentiated carcinoma	2.87	0.97	8.51	0.057
Other subtypes	1.60	0.64	4.02	0.3
Squamous cell carcinoma	1.68	0.94	3.00	0.078
Adenoid cystic carcinoma	0.61	0.26	1.43	0.3
Mucoepidermoid carcinoma	0.79	0.33	1.92	0.6
Salivary duct carcinoma	3.30	1.38	7.94	**0.008**
Acinic cell carcinoma	0.88	0.40	1.92	0.7
Epithelial-myoepithelial carcinoma	0.35	0.08	1.52	0.2
Carcinoma ex-pleomorphic adenoma	1.77	0.80	3.89	0.2
Myoepithelial carcinoma	1.31	0.38	4.52	0.7
Neck dissection				
No	1	Reference		
Yes	0.53	0.35, 0.79		**0.002**
AJCC Stage				
I/II	1	Reference		
III/IV	1.58	1.02, 2.45		**0.039**
**Model 3c: Age, histology, treatment, and traditional staging I**				
Age, median: 66 years				
<Median	1	Reference		
>Median	2.42	1.50	3.90	**<0.001**
Histology				
Adenocarcinoma	1	Reference		
Carcinoma not specified	0.90	0.35	2.32	0.8
Undifferentiated carcinoma	3.28	1.07	10.1	**0.038**
Other subtypes	2.06	0.80	5.28	0.13
Squamous cell carcinoma	1.94	1.04	3.61	**0.036**
Adenoid cystic carcinoma	0.73	0.30	1.79	0.5
Mucoepidermoid carcinoma	0.78	0.32	1.92	0.6
Salivary duct carcinoma	2.70	1.05	6.96	**0.039**
Acinic cell carcinoma	0.86	0.3	1.97	0.7
Epithelial-myoepithelial carcinoma	0.41	0.09	1.81	0.2
Carcinoma ex-pleomorphic adenoma	1.72	0.74	4.02	0.2
Myoepithelial carcinoma	1.59	0.44	5.66	0.5
Neck dissection				
No	1	Reference		
Yes	0.51	0.34	0.79	**0.002**
T classification				
T1/2	1	Reference		
T3/4	1.28	0.81	2.02	0.3
N classification				
N0	1	Reference		
N+	1.34	0.86	2.08	0.2

M classification				
M0	1	Reference		
M1	2.66	1.06	6.69	**0.037**
**Model 4c: Age, histology, treatment, and traditional staging II**				
Age, median: 66 years				
<Median	1	Reference		
>Median	2.51	1.57,	4.03	<0.001
Histology				
Adenocarcinoma	1	Reference		
Carcinoma not specified	0.87	0.34	2.26	0.8
Undifferentiated carcinoma	3.15	1.03	9.65	**0.044**
Other subtypes	2.00	0.78	5.13	0.15
Squamous cell carcinoma	2.01	1.08	3.72	**0.027**
Adenoid cystic carcinoma	0.72	0.30	1.76	0.5
Mucoepidermoid carcinoma	0.80	0.33	1.97	0.6
Salivary duct carcinoma	3.32	1.36	8.11	**0.009**
Acinic cell carcinoma	0.96	0.43	2.13	>0.9
Epithelial-myoepithelial carcinoma	0.41	0.09,	1.79	0.2
Carcinoma ex-pleomorphic adenoma	1.79	0.77	4.18	0.2
Myoepithelial carcinoma	1.70	0.47	6.10	0.4
Neck dissection				
No	1	Reference		
Yes	0.54	0.36	0.82	**0.003**
T classification				
T1/2	1	Reference		
T3/4	1.32	0.84	2.07	0.2
N classification				
N0	1	Reference		
N+	1.37	0.88	2.13	0.2
**Model 5c: Age, histology, treatment, and alternative staging I**				
Age, median: 66 years				
<Median	1	Reference		
>Median	2.25	1.06,	4.78	**0.035**
Histology				
Adenocarcinoma	1	Reference		
Carcinoma not specified	2.43	0.72	8.17	0.2
Undifferentiated carcinoma	1.34	0.17	10.5	0.8
Other subtypes	8.39	2.88	24.5	**<0.001**
Squamous cell carcinoma	4.07	1.74	9.53	**0.001**
Adenoid cystic carcinoma	0.62	0.17	2.20	0.5
Mucoepidermoid carcinoma	0.73	0.22	2.45	0.6
Salivary duct carcinoma	3.90	1.33	11.4	**0.013**
Acinic cell carcinoma	1.34	0.41	4.39	0.6
Epithelial-myoepithelial carcinoma	0.00	0	100	1.00
Carcinoma ex-pleomorphic adenoma	0.60	0.08	4.47	0.6
Myoepithelial carcinoma	46.3	2.83	7.58	**0.007**
Neck dissection				
No	1	Reference		
Yes	1.00	1	1.00	1.00
T classification				
T1/2	1	Reference		
T3/4	2.89	1.48	5.66	**0.002**
LODDS, median −1.23				
<Median	1	Reference		
≥Median	1.24	0.63	2.44	0.5
**Model 6c: Age, histology, treatment, and alternative staging II**				

Age, median: 66 years				
<Median	1	Reference		
>Median	2.01	0.97	4.19	0.062
Histology				
Adenocarcinoma	1	Reference		
Carcinoma not specified	3.04	0.92	10.1	0.069
Undifferentiated carcinoma	1.76	0.46	6.78	0.4
Other subtypes	7.03	1.8	27.2	**0.005**
Squamous cell carcinoma	2.66	1.0	6.82	**0.042**
Adenoid cystic carcinoma	0.53	0.16	1.75	0.3
Mucoepidermoid carcinoma	0.73	0.22	2.40	0.6
Salivary duct carcinoma	3.70	1.03	13.3	**0.045**
Acinic cell carcinoma	1.03	0.31	3.36	1.00
Epithelial-myoepithelial carcinoma	0.22	0.03	1.76	0.2
Carcinoma ex-pleomorphic adenoma	1.11	0.30	4.14	0.9
Myoepithelial carcinoma	15.5	1.57	152	**0.019**
Neck dissection				
No	1	Reference		
Yes	0.44	0.20	0.95	**0.037**
T classification				
T1/2	1	Reference		
T3/4	2.39	1.23	4.61	**0.010**
LODDS-PAR, median −1.11				
<Median	1	Reference		
≥Median	1.32	0.68	2.59	0.4

HR = hazard ratio; CI = confidence interval; LODDS = log odds of positive lymph nodes, neck, ipsilateral; LODDS-PAR = log odds of positive lymph nodes, neck and parotid, ipsilateral. Significant p-values ($p < 0.05$) in bold.
